# Around and beyond 53BP1 Nuclear Bodies

**DOI:** 10.3390/ijms18122611

**Published:** 2017-12-05

**Authors:** Anne Fernandez-Vidal, Julien Vignard, Gladys Mirey

**Affiliations:** Toxalim (Research Centre in Food Toxicology), Université de Toulouse, INRA, ENVT, INP-Purpan, UPS, 31027 Toulouse, France; anne.fernandez-vidal@inra.fr

**Keywords:** 53BP1, nuclear bodies, DNA damage, replication stress, common fragile sites, genetic instability, cancer

## Abstract

Within the nucleus, sub-nuclear domains define territories where specific functions occur. Nuclear bodies (NBs) are dynamic structures that concentrate nuclear factors and that can be observed microscopically. Recently, NBs containing the p53 binding protein 1 (53BP1), a key component of the DNA damage response, were defined. Interestingly, 53BP1 NBs are visualized during G1 phase, in daughter cells, while DNA damage was generated in mother cells and not properly processed. Unlike most NBs involved in transcriptional processes, replication has proven to be key for 53BP1 NBs, with replication stress leading to the formation of these large chromatin domains in daughter cells. In this review, we expose the composition and organization of 53BP1 NBs and focus on recent findings regarding their regulation and dynamics. We then concentrate on the importance of the replication stress, examine the relation of 53BP1 NBs with DNA damage and discuss their dysfunction.

## 1. Introduction

The cell nucleus is the site of key cellular events such as replication, transcription and RNA maturation. Within the nucleus, sub-domains are defined and this spatial organization plays an important role in these specific functions. Nucleoli are the archetype of such sub-nuclear territories, where ribosomal transcription and maturation occur [1], but many other sub-nuclear regions have been described and regrouped under the terminology of nuclear bodies (NBs). One can define NBs as dynamic structures, able to exchange components with the nucleoplasm, and concentrate nuclear factors and that can be observed microscopically [2]. Although the biogenesis mechanisms may differ among NBs, some common aspects have emerged underlining particularly the role of RNAs [3,4]. Among these NBs are Cajal bodies, associated with short non-coding RNA metabolism, histone locus bodies where histone mRNAs are processed, paraspeckles involved in the retention of RNA molecules, etc. [2,3]. 

Unlike NBs involved in RNA metabolism, some NBs are related to the DNA damage response (DDR). This is the case of promyelocytic leukemia (PML) bodies, first identified using autoantibodies from primary biliary cirrhosis patients, then with antibodies against the PML factor [5]. In acute promyelocytic leukemia, PML NBs are perturbed because oncogenic PML-RARα (chimera involving PML and the retinoic acid receptor α) disrupts these structures into multiple dispersed smaller bodies [6,7]. PML NBs are involved in multiple functions, for example cell proliferation, senescence, apoptosis, DNA damage response and repair [8]. PML bodies have been found to contain an accumulation of proteins implicated in these processes, and the disruption of such proteins hamper PML NBs formation. PML NBs are also the sites of protein post-translational modifications, with the formation of PML NBs depending on PML SUMOylation status, among others [8,9,10], and PML NBs being involved in p53 acetylation—although not directly through PML [11,12]. Furthermore, DNA damage leads to an increase of PML NBs [13]. The link between PML bodies and DDR was confirmed in PML mutant cells, as PML-deficient cells present a high level of sister chromatid exchange [14] and fail to activate p53 after DNA damage [11]. Although the role of PML NBs in genome maintenance makes them unique among all NBs [8], another nuclear domain presents singular specificities.

The p53 binding protein 1 (53BP1) is a key component of the DDR that forms microscopically visible foci at double-strand breaks (DSB) sites [15]. A defect in 53BP1 induces DNA damage checkpoint defects, impaired DNA repair and hypersensitivity to ionizing radiation [16]. In addition to these numerous small foci, 53BP1 has also been involved in different large NBs as telomere dysfunction-induced foci (TIFs) [17], DNA segments with chromatin alterations reinforcing senescence (DNA-SCARS) [18] and finally 53BP1 nuclear bodies (53BP1 NBs). The 53BP1 NBs were first described as Oct1/PTF/transcription (OPT) domains [19], formed around DNA lesions generated during mitosis at loci that failed to complete DNA replication. Transmitted to daughter cells, these DNA lesions are sequestered into large chromatin domains enriched in 53BP1 and other markers associated with DDR [20]. Here, we will focus on recent findings regarding 53BP1 NBs, their organization in the nucleus, their regulation and their dynamics. We will also discuss the roles they play in the regulation of cellular processes such as replication stress (RS), DNA damage/repair, cell cycle and their dysfunctions.

## 2. Composition of p53 Binding Protein 1 (53BP1) Nuclear Bodies (NBs)

Many years before their extensive characterization, 53BP1 NBs have previously been observed in several studies. Twenty years ago, Pombo et al. described the focal accumulation of two transcription factors, Oct1 and PTF, in 0.5 to 3.5 μm nuclear area defined as OPT domains [19]. This study already pointed out the dynamic behavior of such structures, formed in G1 phase and lost in early S. Analogous domains containing the ATP-dependent RNA helicase DDX1 (DEAD-box helicase 1), involved in various aspects of RNA processing, have also been described as DDX1 bodies [21]. Apart from these observations, it has long been noticed that 53BP1, a DNA damage response (DDR) protein recruited at DSB sites [15], displays a singular localization pattern as large nuclear speckles in a subset of cells without any exogenous stress [22]. These nuclear structures actually represent the same 53BP1 NBs, containing many factors involved in DNA damage signaling or gene expression regulation [20,23]. The incoming section dresses a list of the different proteins that compose 53BP1 NBs with their cellular functions.

A first class of proteins found in the 53BP1 NBs are dedicated to DSB signaling. Initial steps of cellular response to DSB involve the recruitment of ATM (ataxia-telangiectasia mutated kinase) and the MRN (MRE11-RAD50-NBS1) complex to damaged site, allowing ATM activation through auto-phosphorylation at S1981 [24,25]. ATM then phosphorylates hundreds of targets [26], including H2AX (histone H2A variant), referred to as γH2AX once phosphorylated on S139 [27]. This generates a binding site for the scaffold protein MDC1 (mediator of DNA damage checkpoint protein 1) [28], which in turn drives the recruitment of the E3 ubiquitin-ligase RNF8 [29]. RNF8 initiates a localized K63-linked ubiquitin signaling that promote the recruitment of RNF168, another E3 ubiquitin ligase [30], subsequently propagating the K63 ubiquitin chains around the DNA lesion [31]. These DSB-associated ubiquitin chains finally recruit two major signaling proteins, 53BP1 and BRCA1 (breast cancer 1) [32,33], governing the choice between the two canonical DSB repair processes, non-homologous end joining (NHEJ) and homologous recombination (HR), respectively [34,35,36]. All these DSB signaling factors have been shown to compose 53BP1 NBs [20,23]. Moreover, a previous study suggested that the DSB sensor KU complex might also be part of bodies similar to 53BP1 NBs [37]. Altogether, these observations imply that 53BP1 NBs might represent sites of damaged chromatin, confining the signaling of one or more DSBs in a defined nuclear domain. 

Conversely, proteins that are directly involved in DSB repair mechanisms or in RS response are largely excluded from 53BP1 NBs. These include the two HR factors CtIP and RAD51, the early transductors of RS signaling ATR (ataxia telangiectasia and Rad3-related) and ATRIP (ATR interacting protein), and the Fanconi anemia (FA) protein FANCD2 (Fanconi anemia group D2 protein) [23]. The single-stranded DNA (ssDNA) binding protein RPA (replication protein A), a key player in HR process and RS response, is mainly absent from 53BP1 NBs, albeit the proportion of RPA positive NBs can be experimentally increased when replication origin number is altered (see below) [38]. Another regulator of HR and replication fork stability, the RecQ helicase BLM (Bloom syndrome protein) [39], is also present in only a subset of 53BP1 NBs [23]. Apart from these factors lies the scaffold protein TopBP1 (DNA topoisomerase 2-binding protein 1), which participates in several pathways: replication initiation by the binding of proteins mediating the loading of CDC45 on origins; DNA checkpoint signaling by stimulating the full activation of ATR following replication stress; DNA repair by orchestrating repair pathway choice (HR or NHEJ) and regulation of transcription following the binding of several transcription factors [40]. TopBP1 strongly colocalizes with 53BP1 NBs [23,41]. 

Behind the signaling of DSB, 53BP1 NBs appear to locally regulate transcription. The presence of Oct1 and PTF transcription factors have first been considered as a mark of enhanced transcription [19]. However, the exclusion from 53BP1 NBs of the initiating and elongation forms of RNA Pol II, and the lack of 5-fluorouridine or 5-ethynyl uridine staining, two markers of active transcription sites, strongly suggest that transcription is actually repressed in 53BP1 NBs [20,42]. This is in agreement with transcription silencing at DSB sites that is strictly dependent on ATM and partially on RNF8 and RNF168 [43]. Besides, several RNA processing factors have also been visualized at 53BP1 NBs. As mentioned above, the RNA helicase DDX1 has been shown to be a component of these nuclear domains [20,21]. Moreover, the alternative splicing regulator PPM1G (protein phosphatase 1G), a nuclear member of the PP2C family of Ser/Thr phosphatases, and the RNA/DNA helicase senataxin that removes R-loop during transcription termination, are also enriched at 53BP1 NBs [44,45]. The presence of these proteins at 53BP1 NBs may not rely on their role in transcription regulation but rather reflect a more general role in DDR [44,45,46,47,48].

**Figure 1:**
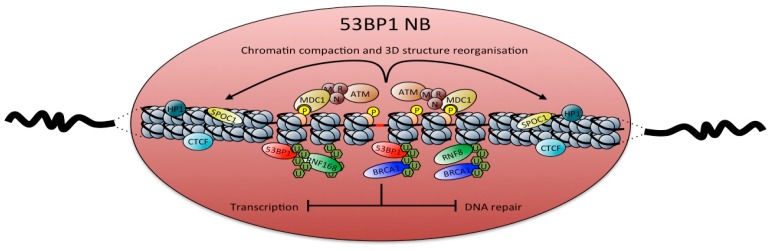
Composition of 53BP1 nuclear bodies. Unreplicated DNA from mother cell (red area) induces the accumulation of DDR signaling factors in G1 daughter cells (53BP1 (red), MDC1, MRN complex, ATM (brown), RNF8, RNF168 (green) and BRCA1 (blue), associated with histone H2A/H2AX ubiquitinylation (green U chains) and H2AX phosphorylation, i.e., γH2AX (yellow circled P on nucleosomes). This in turn locally alters chromatin structure through the recruitment of the insulator protein CTCF (light blue) and enhances chromatin compaction mediated by SPOC1 (yellow) and HP1 (blue). Exclusion from 53BP1 NBs of RNA Pol II and DNA repair factors locally inhibits transcription and DNA repair, respectively.

Finally, local changes in chromatin structure seem to be associated to 53BP1 NBs. This is exemplified by the accumulation to such domains of CTCF (CCCTC-binding factor), an insulator protein that establishes functional three-dimensional chromatin structures [49]. Factors influencing chromatin compaction are also present at 53BP1 NBs. These include SPOC1, which modulates the association of many heterochromatin-building factors as HP1 (heterochromatin protein 1), also present at 53BP1 NBs [50,51]. On the other hand, 5-hydroxymethylcytosine (5hmC), a modified DNA base associated to open chromatin structure, also localizes at these NBs [52]. According to the drastic changes in chromatin configuration at DNA damage sites [53] or to growing body of evidences of 5hmC roles in DNA repair [54], modification of chromatin structure at 53BP1 NBs should be a consequence of DDR activation. However, while 5hmC accumulation signals an open chromatin state necessary for efficient DNA repair, SPOC1-mediated chromatin compaction has been correlated to NHEJ repression [51]. Therefore, 53BP1 NBs integrity should require an open chromatin environment to efficiently signal DNA damage. At the same time, transcription is tightly regulated and DNA repair may be inhibited, or more probably just delayed until a more favorable cellular context (Figure 1).

## 3. Dynamics of 53BP1 NBs

53BP1 NBs display characteristics making them unique among the other nuclear domains. They are located in independent area of the nucleus compared to other NBs, except for PML that are often present at the periphery or contained within 53BP1 NBs [19,20,55]. PML also regulates the local motion of 53BP1 NBs [56]. 

The most striking feature of 53BP1 NBs is their cell cycle-dependent integrity. Whereas other NBs are present all over the interphase, 53BP1 NBs visualization is restricted to G1/G0. Interestingly, 53BP1 NBs formation upon mitosis exit frequently occurs in a symmetrical manner between the two daughter cells, strongly suggesting that these domains derive from stresses induced at the previous cell generation [20,23,57]. Knowing that 53BP1 NBs result from replication defects that arise during the precedent parental S phase (see below for details), their formation at the next G1 phase raises the issue of the temporal shift between a stress experienced by a mother cell and its signaling in daughter cells. 

53BP1 is a bivalent histone mark reader, requiring both the recognition of H4K20me2 by its tandem Tudor domains and the binding of RNF168-induced H2AK15 ubiquitylation through its UDR (ubiquitination-dependent recruitment) motif, to properly accumulate at DSB sites during the classical DDR [32,58]. However, 53BP1 and other late DSB signaling factors, namely RNF8, RNF168 and BRCA1, are unable to recognize chromatin during mitosis, and their loading to damaged chromatin is delayed until the next G1 [59]. Indeed, mitotic kinases CDK1 (cyclin-dependent kinase 1) and PLK1 (polo-like kinase 1) phosphorylate RNF8 and 53BP1 to prevent their recruitment to DSB sites and the consecutive deleterious DSB repair that may cause telomere fusion [60]. Conversely, the PP4C/R3b phosphatase complex dephosphorylates 53BP1 to alleviate inhibition of chromatin association and permit 53BP1 NB formation, at the onset of G1 or even before the G1 phase, i.e., during cytokinesis [61,62]. On the other hand, live cell imaging indicates that the early DSB signaling factor MDC1 forms nuclear foci on mitotic chromatin that are transmitted to daughter cells, strongly supporting that γH2AX, the MDC1 binding site, is also conserved from mitosis to G1 [23]. Remarkably TopBP1, that possesses multiple BRCT (BRCA1 C terminus) domains driving its recruitment to sites of replication stress in S/G2 or DSBs in G1 by distinct mechanisms [63], also forms foci after mitotic entry that gradually disappear as mitosis progresses, and transition into 53BP1 NBs [41]. These data confirm that 53BP1 NBs mark chromatin sites that were already damaged at previous mitosis but could not be properly repaired.

Finally, 53BP1 NBs progressively disappear during replication, the majority of which being processed at early S [20,23,38]. This may be partly due to replication-coupled dilution of H4K20me2 that reduces 53BP1 binding to post-replicative chromatin [64]. However, the cellular mechanisms governing 53BP1 NBs resolution have not been precisely investigated so far. The presence of BRCA1 at these domains [23] raises the question of the potential role of HR to overcome the cellular stresses signaled by 53BP1 NBs. BRCA1 focus formation in response to irradiation-induced DSBs is usually inhibited in G1 by the 53BP1-RIF1 (replication timing regulatory factor 1) axis [65]. Conversely, BRCA1 recruitment to DSBs prevents RIF1 accumulation and therefore directs repair toward HR. In accordance with HR inhibition in G1 to prevent loss of heterozygosity [66], HR factors CtIP and RAD51 are not present in 53BP1 NBs [23]. It is, therefore, tempting to speculate that BRCA1-containing NBs in G1 prime damaged loci to be later processed by HR mechanisms at the next S phase.

## 4. Replication Stress and 53BP1 NBs

### 4.1. Sources of Replication Stress (RS)

The DNA replication machinery must carry out faithful, complete and speedy duplication of genetic information to preserve genome stability and elicit correct chromosome segregation in mitosis. Replication forks (RFs) are continuously challenged by numerous intracellular and extracellular impediments, leading to fork slowing down or stalling termed replication stress (RS) [67,68]. In addition to direct DNA lesions, RS can be caused by naturally difficult to replicate loci due to inherent characteristics, as chromatin compaction, repetitive regions, secondary structures, DNA/RNA hybrids, collision between replication and transcription machineries, etc. Limitation of factors essential for DNA replication, such as modification of dNTPs pool or deregulation of replication origins licensing and firing, also results in RS. Finally, oncogene activation or tumor suppressor inactivation may also facilitate RS by promoting G1/S transition or stimulating global transcription and drive chromosomal instability (CIN) [69,70,71,72].

### 4.2. 53BP1 NBs Originate from RS

The initial finding that silencing of several factors involved in various aspects of DNA replication—such as ATR, CHK1, TopBP1, MCM10, PolA, PolE, RPA, TOP2A, PALB2, BRCA2, RAD51-increases the number of 53BP1 NBs, suggests that RS could be the cause of 53BP1 NBs formation [23]. This has been confirmed by the 53BP1 NBs increase observed after aphidicolin treatment, a natural inhibitor of replicative DNA polymerases that perturbs DNA replication at distinct loci called fragile sites (see below), at low doses, without noticeably affecting cell cycle progression [20,23]. RS triggered by the depletion of PIGN, MEX3C or ZNF516, three CIN-suppressors in colorectal carcinogenesis, induces 53BP1 NBs assembly [69]. Altering the number of replication origin also modulates 53BP1 NBs, with the reduction of replication origins —after MCM4, MCM5 or CDT1 depletion—favoring under-replicated regions between two stalled forks and subsequently generating 53BP1 NBs, while overexpression of CDC6 and enhancement of origin numbers reduces 53BP1 NBs [38,73].

Despite RS constitutes the major source of 53BP1 NBs, ssDNA seems to be excluded or rarely localize in these structures (with RPA < 1% of 53BP1 NBs after treatment with aphidicolin) [20,23]. However, Blow’s team highlighted a weak RPA association with 53BP1 NBs (7%) in G1 untreated HeLa cells [38]. Following a reduction of replication origins, due to a partial depletion of MCM2–7, 30% of 53BP1 NBs contained RPA, suggesting that fork stall occurring naturally in large replicon or after origins depletion generates under-replicated DNA region, at least partially loaded by RPA in G1 cells. In this context, no γH2AX variation was observed in G1 cells after MCM5 depletion. This suggests that 53BP1 NBs are complex bodies probably containing DSBs and/or other DNA structures, yet protected until another round of replication allow their resolution.

### 4.3. Response to RS Regulates 53BP1 NBs

Resolving RS is crucial to maintain genome stability and DDR constitutes a major anti-tumor barrier [74,75,76]. Interestingly, unperturbed growing medulloblastoma cell lines or sample from patients prior to therapy display elevated 53BP1 NBs, scars of RS in the previous cell cycle [76]. ATR, the master mediator in response to RS, controls the replication fork progression, prevents its collapse, inhibits new origins firing and assists the restart of stalled forks. Therefore, ATR deficiencies have been correlated with 53BP1 NBs induction [20,23,77] while CHK1 kinase—a major ATR target involved in checkpoint activation and RS rescue-prevents 53BP1 NBs [23]. Recently ETAA1 (Ewings tumor-associated antigen 1), a new factor contributing to ATR activation independently of TopBP1, has been identified; both TopBP1 and ETAA1 contribute to reduce 53BP1 NBs [41,78], but their epistasis relationship has not been investigated so far. Besides, the FA/HR is a major pathway to stabilize, protect from deleterious resection and rescue RF progression. Three FA proteins, FANCA, FANCC and FANCM, were shown to prevent 53BP1 NBs [73,79,80,81]. Moreover, BOD1L, a newly identified FA-related factor that inhibits RF over-resection after RS, has also been shown to attenuate 53BP1 NB formation [80]. Finally, in FA deficiency, HELQ (Helicase POLQ-like) seems to assist in the stalled fork recovery during unperturbed S phase, therefore limiting the spontaneous 53BP1 NBs appearance [81]. Taken together, these results highlight the role of RF protection mechanisms to prevent RS-mediated 53BP1 NBs.

## 5. Common Fragile Sites (CFS) and 53BP1 NBs

Common fragile sites (CFS) are present in all individuals and are defined as chromosomal regions prone to breakage when cells are submitted to moderate fork slowing, such as treatment with low doses of aphidicolin [82]. Expression of CFS—meaning breaks, gaps or rearrangements of fragile sites—constitutes the main source of genome instability in pre-neoplastic lesions [83]. CFS exhibit several common characteristics that could contribute together to their fragility: they are nested in very large genes (ranging from 0.6 Mb to more than 2 Mb in length) generating long primary transcripts, replicated in late S phase and display large initiation-poor regions associated to condensed chromatin. Moreover, CFS show high propensity to form DNA secondary structures within AT-rich sequences, impeding fork progression [84].

Chromatin immuno-precipitation (ChIP) experiments revealed an enrichment of 53BP1 on CFS (FRA16D and FRA3B loci) after low dose of aphidicolin [23,85] and, under mid-RS, 19% of 53BP1 NBs are positive for FRA3B [86]. Additionally, γH2AX enrichment was shown on many CFS, by ChIP-sequencing, in G0-arrested BJ hTERT (immortalized) fibroblasts without any treatment [20]. These data support that 53BP1 NBs originate from lesions caused by RS, preferentially accumulating at CFS. 

Several specialized DNA polymerases were described to be involved in CFS stability. The Rev3 catalytic subunit of Pol ζ, Pol η and Pol κ could facilitate DNA synthesis through CFS [87,88,89]. Consequently, their depletion leads to 53BP1 NBs in the G1 daughter cells: unperturbed cells deficient for *REV3L* exhibit NBs in G1, containing 53BP1 and γH2AX [90] and, in mid-RS and absence of Pol η, under-replicated DNA appears at CFS, generating 53BP1 NBs in G1 daughter cells [87]. Moreover, the RAD18-dependent SUMOylation of Pol η by the SUMO ligase PIAS1 is required to control Pol η recruitment to CFS, preventing chromosome fragmentation in mitosis and 53BP1 NBs accumulation in G1 [91]. Concerning Pol κ, specialized DNA polymerase playing a role in the recovery of stalled fork by facilitating checkpoint activation, its deficiency induces 53BP1 NBs, further exacerbated by aphidicholin treatment [92]. However, Pol κ recruitment on chromatin needs to be tightly regulated. Indeed, an original function of p21 was recently proposed, in absence of DNA damage, to prevent unscheduled loading of Pol κ on undamaged templates in order to avoid RS, CFS instability, 53BP1 NBs assembly and micronuclei formation [93]. The deubiquitinase USP1 also plays a key role in Pol κ recruitment to the replication fork in absence of external stress, and USP1-depleted cells display elevated 53BP1 NBs, probably reflecting the genomic instability caused by the hyper-recruitment of Pol κ and/or the aberrant accumulation of mono-ubiquitinated FANCD2 at chromatin [94,95]. 

All these results sustain the emerging concept whereby CFS expression is the major source of 53BP1 NBs. Indeed, RS induced by hydroxyurea—another drug that stall RFs without affecting specifically CFS [82,96]—is not associated with 53BP1 NBs accumulation, unlike aphidicolin [20]. However, we cannot exclude that other genomic regions could also constitute natural replication fork barriers, promoting under-replicated loci transmitted in G1 to daughter cells and sequestered in 53BP1 NBs. In fact, Harrigan et al. discovered several regions of γH2AX accumulation located on chromosomes 1, 9, 20 and 21 of G0 arrested cells, which do not match with CFS [20] and may constitute other natural replication-fork obstacles (see above, Section 4.1). 

## 6. Late DNA Synthesis for Under-Replicated Regions

### 6.1. Mitotic DNA Synthesis (MiDAS)

Intrinsically difficult to replicate DNA regions (such as CFS, large replicons, etc.) generate under-replicated DNA, escape checkpoint activation and reach mitosis. However, a mitotic DNA synthesis (MiDAS) mechanism has recently been elucidated to overcome such replication failures before metaphase onset [97]. Following mid-RS, under-replicated CFS of early mitotic cells are recognized by FANCD2 that forms “twin foci” on each sister chromatid [98,99]. Then, the sequential recruitment of the scaffold protein for nucleases and SUMO E3 ligase SLX4, the chromosome condensation inducer SMC2 (structural maintenance of chromosomes protein 2), the regulator of sister-chromatin arm cohesion WAPL and finally the ssDNA annealing protein RAD52 promote the recruitment of MUS81-EME1, XPF-ERCC1 and GEN1 endonucleases on these persistent replication intermediates [85,86,100,101,102,103]. The presence of MUS81 on CFS leads to RecQ5 helicase recruitment that, in turn, facilitates MUS81 endonuclease activity by dismantling RAD51 filaments from the fork [104]. CDK1 plays a deciding role in the coordination of all these actors by phosphorylation of EME1 and RecQ5 at the onset of mitosis [97,105]. Thereby these endonucleases generate gaps (DAPI negative) or breaks on metaphase chromosomes that are at least partially filled by the Pol D3 polymerase [97]. These studies suggest that MiDAS occurs via a microhomology-mediated break-induced replication (BIR) mechanism, as it follows a conservative model and involves POLD3 and RAD52, independently of RAD51 [100]. Therefore SLX1, SLX4, SMC2, WAPL, EME1, RAD52, MUS81, ERCC1, GEN1 or POLD3 depleted cells exhibit an increase of 53BP1 NBs in the following G1 [85,86,97,100,101,102,103,104,106]. Finally, TopBP1 also plays an unanticipated and crucial role in mitosis by preventing transmission of DNA damage to daughter cells. TopBP1 partially co-localizes with FANCD2 foci to under-replicated domains in early mitosis after mid-RS, elicits the recruitment of SLX4 and facilitates unscheduled DNA synthesis, avoiding the formation of 53BP1 NBs in G1 [41].

### 6.2. Anaphase Bridges Are the Last Opportunity to Properly Segregate DNA

**Figure 2:**
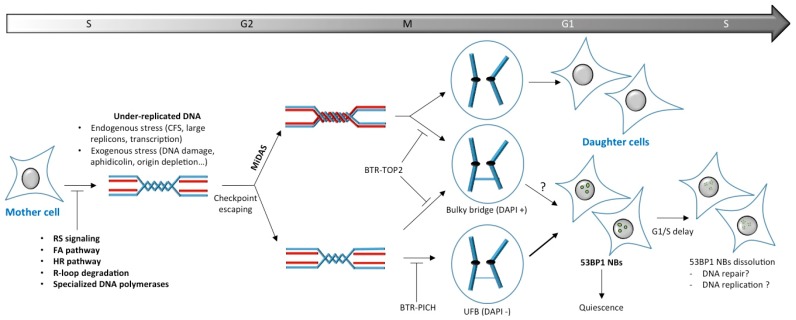
General scheme of 53BP1 NBs formation. During S phase, difficult to replicate loci such as common fragile sites (CFS) and direct DNA damage induce physiological replication stresses responsible of under-replicated DNA, allowing cells to escape checkpoint activation and reach mitosis. As cells enter mitosis, SLX4-MUS81-EME1 complexes cleave under-replicated loci to initiate mitotic DNA synthesis (MiDAS). In absence of sister chromatids decatenation by BLM-TopoIII-RMI1-RMI2 (BTR complex)-TOP2 at MiDAS sites, bulky anaphase bridges (DAPI+) are formed. Incomplete resolution of late replication intermediates through MiDAS induces bulky bridges and DNA ultra-fine bridges (UFBs) formation at anaphase. UFBs resolution by BTR-PICH constitutes the last chance for rescuing partially replicated DNA stretches before the end of anaphase. Impairing faithful resolution of UFBs leads to 53BP1 NBs in G1 daughter cells, whereas a direct link between bulky bridges and 53PB1 NBs is controversial (arrow with question mark). Alternatively, under-replicated regions that are not associated to UFBs may also generates 53BP1 NBs. 53BP1 NBs-positive daughter cells enter in quiescence and/or exhibit a prolonged G1 before reaching S phase. 53BP1 NBs dissolution occurs in early-mid S phase through a process that still need to be elucidated. T bars on the scheme mean that the process is negatively regulated.

DNA ultra-fine bridges (UFBs) arise when a cell enters anaphase without having resolved late replication intermediates through MiDAS. UFBs are labeled by FANCD2/FANCI proteins at their extremities [98,99,107] and the persistence of FANCD2 foci from S/G2 phases to anaphase structures supports that these bridges originate from under-replicated regions. Actually a subclass of UFBs comes from CFS loci and is often γH2AX positive. UFBs constitute the last opportunity to ensure proper sister-chromatid disjunction. The PICH (PLK1-interacting checkpoint helicase) DNA translocase associates along UFBs to prevent DNA bridges and contribute to RIF1 and BLM helicase recruitment that, in concert with Topoisomerase II activity, resolve these bridges during anaphase by decatenation [108]. Thereby, as a consequence of incomplete resolution of these DNA structures, downregulation of BLM, PICH or RIF1 enhances 53BP1 NBs formation in G1 daughter cells. Interestingly, delaying UFB resolution by using topoisomerase 2 inhibitor is sufficient per se to enhance 53BP1 NBs formation, independently of any apparent DNA replication defects [108]. This suggests impairing the faithful resolution of catenated DNA molecules or late replication intermediates in anaphase could give rise to DNA lesions sequestered in 53BP NBs. Therefore, UFB resolution constitutes the last chance for rescuing partially replicated stretches of DNA before the end of anaphase, ensuring genomic stability (Figure 2).

## 7. Major Cellular Processes and 53BP1 NBs

### 7.1. Transcription

As stated earlier, transcription is inhibited at 53BP1 NBs. This regulation is controlled by TRIP12 and UBR5, two HECT domain E3 ligases limiting RNF168 accumulation to 53BP1 NBs, and therefore modulating transcription silencing imposed by DNA damage-induced histone ubiquitylation [42]. In turn, deregulated transcription can generate 53BP1 NBs. For instance, global transcription augmentation in cells overexpressing oncogenic HRAS^V12^ or the TBP general transcription factor induces 53BP1 NBs [109]. In such contexts, 53BP1 NBs increase is a consequence of RNA/DNA hybrids (R-loops) accumulation. R-loop structures generate replication stress by impeding replication fork progression [110]. Indeed, HRAS^V12^- or TBP-induced 53BP1 NBs can be alleviated by RNaseH1, a R-loop degrading enzyme. In the same way, loss of the putative RNA/DNA helicase senataxin, or treatment with the topoisomerase I inhibitor diospyrin D, both induce R-loop formation and increase 53BP1 NBs [45].

### 7.2. Double-Strand Breaks (DSB) Signaling and DNA Repair

Many players of DSB signaling accumulate in 53BP1 NBs. Consequently, alterations in this pathway affect 53BP1 NBs integrity. 53BP1 protein and its NUP153-dependent nuclear import are crucial to constitute these NBs [23,111]. ATM inhibition or depletion also results in a drastic reduction of 53BP1 accumulation at such domains [20,23]. In the same way, down-regulation of ATMIN (ATM interactor), an ATM co-factor required for its activation, affects 53BP1 NBs formation [112]. H2AX and RNF168, two other DSB signaling proteins present in 53BP1 NBs, are essential for these domains to be fully formed [23,112]. On the contrary, MRN deficiency induces an increase of 53BP1 NBs, probably as a consequence of replication stress experienced by cells with impaired MRN complex [23,112,113]. Finally, impairing mitotic DSB signaling alters subsequent 53BP1 NBs formation. Wip1, a PP2C family serine/threonine phosphatase that attenuate DSB signaling by dephosphorylating many proteins including ATM and γH2AX, is normally inhibited in mitosis. Thus, ectopic expression of Wip1 decreased γH2AX on mitotic chromosomes and ensuing 53BP1 NBs [114], establishing mitotic γH2AX positive loci as marks for the future NBs.

Proteins involved in different DNA repair pathways have been shown to regulate 53BP1 NBs formation. This is well exemplified with HR, as depletion of BRCA1, BRCA2, RAD51, PALB2, MRE11 or the RAD51 paralogs XRCC2, XRCC3 and RAD51C, induces an increase of 53BP1 NBs in unstressed cells [23,113,115,116,117]. HR factors also play a distinct role in protecting stalled replication fork from MRE11-dependent degradation of nascent DNA, which could support their role in 53BP1 NBs inhibition [118,119]. However, it has recently been shown that the BRCA2-mediated inhibition of 53BP1 NBs formation relies on its role in HR rather than in replication fork protection [115]. Thus, DNA repair per se could protect cells from accumulating 53BP1 NBs. As mentioned above, the mitotic DNA repair function of the endonucleases MUS81-EME1, XPF-ERCC1, SLX1-SLX4 and GEN1, required to promote mitotic DNA synthesis and chromosome disjunction, are essential to inhibit 53BP1 NBs [85,86,97,103]. Another endonuclease, FEN1, involved in Okazaki fragments processing during replication and in Base Excision Repair, also prevents 53BP1 NBs through its nucleolytic activity [120]. Besides, cells overexpressing FEN1 or XPG, an endonuclease involved in Nucleotide Excision Repair, accumulate 53BP1 NBs [121], confirming that endonucleases must be tightly regulated to avoid 53BP1 NBs due to uncontrolled DNA stand breaks. Finally, deficiency in SPARTAN, protease involved in replication-coupled DNA-protein crosslink repair [122], results in 53BP1 NBs increase [123]. Thus, different replication fork-blocking lesions can subsequently contribute to 53BP1 NBs at the next cell generation.

### 7.3. Chromatin Structure and Chromosome Segregation

Similarly to DSB signaling factors, modulators of chromatin conformation that are present at 53BP1 NBs also regulate these structures. Indeed, CTCF- and HP1-deficient cells accumulate more 53BP1 NBs than control cells [49,50]. In both cases, 53BP1 NBs increase may be a consequence of HR defects, as CTCF and HP1 promote RAD51 and BRCA1 recruitment to DSBs, respectively. On the other hand defect in 5hmC production, after depletion of TET enzymes, induces replication stress-related chromosome missegregation associated with a decrease in 53BP1 NBs formation [52]. Moreover, global mitotic chromosome compaction by condensin complex is crucial for 53BP1 NB formation, but the precise role of condensin proteins is still under debate. Indeed a first study showed that depletion of SMC2 enhances UFBs and reduces 53BP1 NBs [23], while a second demonstrated that loss of SMC2 resulted in 53BP1 NBs increase, accompanied with UFBs increase due to MUS81 recruitment failure that prevented MiDAS [97]. 

Chromosome segregation defects represent another putative route to generate 53BP1 NBs. Impairing kinetochore-microtubule attachment, with monastrol or by inhibiting the mitotic checkpoint kinase Mps1, provokes chromosome missegregation followed by 53BP1 NBs formation in daughter cells [124]. This response can be reverted by blocking cytokinesis, with inhibitors of myosin II or Aurora B, suggesting that 53BP1 NBs originated from breakage of missegregated chromosome at cleavage furrow. In contrast, RS-induced NBs do not depend on cytokinesis [23]. However, another study depicted the relationship between mitotic errors and subsequent replication stress to be a source of 53BP1 NBs [125]. Notably, this work demonstrates that depletion of ANLN, a major cytokinesis regulator, progressively increased 53BP1 NBs at the next G1 phases where daughter nuclei reside in the same cytoplasm due to cytokinesis failure, while harboring RS hallmarks during S phase. Overall, these findings emphasized mitotic stresses as a cause of inherited damage, and thus of 53BP1 NBs.

### 7.4. Connection with the Cell Cycle

53BP1 NBs are closely connected to the cell cycle, with cell cycle alterations modifying the 53BP1 NBs formation and reciprocally. For example, silencing of genes involved in the cell cycle checkpoints led to 53BP1 NBs increase [23]. In the same way, inhibition of WEE1, a cell cycle regulating kinase, enhances CDK activity and uncontrolled replication initiation, followed by RS and 53BP1 NBs formation in the next G1 phase [126]. Furthermore, a link may exist between the length of cell cycle phases and 53BP1 NBs. The important proliferation rate of Embryonic Stem Cells (ESCs) imposes rapid cell cycle gap phases (G1 and G2), associated with high RS and constitutively active DDR, rendering ESCs dependent for replication-coupled DNA repair to maintain genomic integrity [127]. However, these cells only present few 53BP1 NBs. Conversely, differentiated ESCs show longer gap phases and suffer less RS, while harboring more 53BP1 NBs, which is counter-intuitive. In this context, the G1 length seems crucial, because delaying the G1/S transition in ESCs with cell cycle inhibitors allows formation of 53BP1 NBs and RS decrease. Inversely, shortening of the G1 phase in differentiated cells through FZR1 depletion prevents 53BP1 NBs formation and induces RS. Besides, lengthening of G2 phase or mitosis, by transient treatment with a CDK1 inhibitor or with nocodazole respectively, diminishes the number of 53BP1 NBs at the next G1, especially those related to under-replicated DNA due to HR defects [115]. 

In turn, 53BP1 NBs modulate the cell cycle. The fact that 53BP1 NBs have been found in quiescent primary human fibroblasts suggests a correlation between these bodies and cell cycle control. The consequences of 53BP1 NBs for proliferation or quiescence cell fate decision have only been elucidated recently. First, 53BP1 NBs induced by BRCA2 deficiency trigger G1 arrest and cellular senescence in a p53-dependent manner [115]. After mitosis, cells associated to an elevated CDK activity and low p21 level progress through G1, whereas a subset of cells with low CDK activity and high level of p21 enters quiescence [128,129,130]. Using live cell imaging, a correlation appeared between the presence of 53BP1 NBs after RS and a p53-mediated increase of p21, with high p21 levels inducing CDK2 inhibition and G1 arrest, while low p21 levels allow high CDK2 activity and S phase entry [57,131]. In the opposite, p53-defective cells harboring 53BP1 NBs may enter S phase with unresolved DNA lesions, therefore potentially promoting cancer progression [132], although a p53-independent mechanism might exist [85]. In p53-proficient cells, the extent of inherited damage signaled by 53BP1 NBs determines p21 levels, CDK2 activity and the time spent in quiescence. Yet, while this G1 arrest after damage is p21-dependent and the time spent in G1 is proportional to p21 level, the heterogeneous G1 duration in proliferative cells does not depend on p21 [57]. Indeed cycling cells with a delayed G1 can still be observed in absence of p21 but not of p53. Thus, inherited DNA lesions from previous S phase, which are signaled by 53BP1 NBs, induce a characteristic p53-dependent cellular response in the next G1, modulating p21 expression to control CDK2 activity and block S phase entry. Table 1 and Table 2 summarize the genes involved in the increase or decrease of 53BP1 NBs, respectively.

## 8. 53BP1 in Different Types of NBs

Behind 53BP1 NBs, many similar nuclear domains containing 53BP1 have been described to indicate other type of genotoxic insult. Interestingly, all these 53BP1-related bodies have been observed in the context of delayed or arrested cellular events. As mentioned above, 53BP1 NBs can induce G1 arrest. This response is reminiscent to that elicited during senescence, a phenomenon by which cells cease to divide. Whereas replicative senescence is the result of telomere shortening, ultimately triggering the DDR, cellular senescence can be induced via DNA damage or activation of oncogenes, independently of telomere length [133]. During replicative senescence, telomere shortening or uncapping induces the formation of telomere dysfunction-induced foci (TIFs) composed by DDR proteins found in 53BP1 NBs, such as 53BP1, ATM, γH2AX, MRN or MDC1 [17,134]. In cellular senescence, nuclear structures with persistent DDR proteins have been described and called DNA-SCARS for DNA-segments with chromatin alterations reinforcing senescence [18]. As 53BP1 NBs, DNA-SCARS are associated with PML NBs and accumulate DDR proteins (53BP1, ATM, γH2AX, MDC1, MRE11, NBS1), but lack some characteristics of transient, reversible DNA damage foci like DNA repair proteins RPA and RAD51, ssDNA and active DNA synthesis. Besides, TIFs are generally found associated with DNA-SCARS, and they may be considered as DNA-SCARS localized to telomeres. Until recently, the presence at DNA-SCARS of persistent DSBs, resistant to endogenous DNA repair activities, was still debated. A method called DI-PLA (DNA damage in situ ligation followed by proximity ligation assay), allowing the detection and imaging of DSBs in cells, actually showed that senescent cells hold unrepaired DSBs associated with DDR markers [135]. Thus, it appears that these 53BP1-containing nuclear domains tend to signal irreparable DNA lesions (deficient telomere or DSB) that trigger a permanent cell cycle arrest. On the other hand, similar nuclear structures have been observed in G1 cells with clustered DSBs located in actively transcribed genes that have to be processed through HR [136]. In this case, cells still proliferate but delay DSB repair until next S phase to avoid deleterious NHEJ events, reflecting 53BP1 NB dynamics. Therefore, a general role of 53BP1-positive nuclear bodies seems to mark DNA lesions whose repair is problematic, either because of irreparable damage, triggering cell cycle arrest or even senescence, or when the cellular context is not favorable, as for G1 cells requiring proficient HR.

## 9. Conclusions

In recent years, 53BP1 NBs have been widely studied in term of composition, regulation and dynamics. These particular nuclear domains, specifically formed in G1 cells, derive from inherited DNA lesions in mother cells, and more precisely with under-replicated DNA. However, the molecular processes governing their resolution, probably during the ensuing S phase, still need to be elucidated. More generally, replication stress contributes to human diseases, particularly to genetic instability and cancer progression [137,138]. Recent reviews also examined the link between replication stress and other diseases as neurodegeneration, growth defects and aging [68,139,140], however without assessing the status for 53BP1 NBs in such diseases.

Nonetheless, few publications reported the formation of 53BP1 NBs in the context of specific diseases. Ataxia with oculomotor apraxia 2 (AOA-2) is a neurodegenerative disorder caused by defective senataxin, RNA/DNA helicase to resolve R-loop structures at transcription pause sites. Interestingly, senataxin associates to 53BP1 NBs in response to impaired DNA replication, and senataxin deficiency induces 53BP1 NB formation [45]. Intriguingly, 53BP1 NBs have been described in cancer cells, for example in advanced human papillomavirus (HPV)-positive cervical cancer cells [141]. In addition, tumors associated to transforming viruses like HPV have been found positive for 53BP1 NBs, compared to HPV negative tumors [42]. This led the authors to suggest that the formation of 53BP1 NBs could be a strategy for the viruses to subvert the cell mechanism in order to expand the host cells lifespan and sustain the viral infection. Finally, 53BP1 NBs are also observed in medulloblastoma cells and denote the chronic endogenous replication stress existing in these cells, often infected with human cytomegalovirus [76]. Remarkably, 53BP1 NBs are mainly observed in a non cell-autonomous manner, that is to say in tumor cells that are not infected with the virus but are close to infected cells, within the tumor.

To conclude, a global view on 53BP1 NBs formation is emerging and is intrinsically connected to replication stress. However, 53BP1 NBs may still hide some molecular complexity in order to explain the cellular outcomes, particularly regarding such different diseases as neurodegenerative disorders and cancers. Undoubtedly, future research regarding the consequences of replication stress in the context of different pathologies will shed light on cell regulation and may at the same time uncover new cell mechanisms.

## Figures and Tables

**Table 1:** Genes involved in increasing p53 binding protein 1 (53BP1) nuclear bodies (NBs) when misregulated.

Misregulation	Gene	Functions Related to 53BP1 NBs Regulation	References
RNAi	*ANLN*	Cytokinesis regulation	[125]
RNAi; KO	*ATMIN*	ATM activation	[112]
RNAi; inhibitor	*ATR*	Kinase; RS	[20,23,77]
RNAi	*BLM*	RF stability and restart; HR	[23,108]
RNAi	*BOD1L*	RF protection	[80]
RNAi	*BRCA1*	HR regulation	[116]
RNAi; KO	*BRCA2*	HR, protection of stalled RF	[23,106,115]
RNAi	*CDKN1A*	Checkpoint protein	[93]
RNAi	*CDT1*	Initiation of replication	[38]
RNAi	*CHK1*	RS signaling	[23]
RNAi	*CTCF*	Insulator protein; regulator of chromatin structure	[49]
RNAi	*EME1*	Regulatory subunit of the MUS81-EME1 nuclease; RF restart, HR, MiDAS	[86,97]
RNAi	*ERCC1*	Regulatory subunit of the ERCC1-XPF nuclease; NER, ICL repair, SSA, HR	[86]
KO	*ETAA1*	ATR activation	[78]
RNAi	*FANCA*	FA pathway	[80]
KO	*FANCC*	FA pathway	[73,81]
RNAi	*FANCM*	FA pathway	[79]
RNAi	*FBXO5*	Ubiquitin ligase complex; mitotic regulator	[23]
Overexpression, L209P substitution	*FEN1*	Nuclease; BER, processing of Okasaki fragments	[120,121]
RNAi	*GEN1*	Resolvase; HR	[103]
RNAi	*HP1*	Heterochromatin factor	[50]
Overexpression	*HRAS*	Control of cell proliferation	[109]
F345I substitution	*MCM4*	Initiation of replication	[73]
RNAi	*MCM5*	Initiation of replication	[38]
RNAi	*MCM10*	Initiation of replication	[23]
RNAi	*MEX3C*	RNA-binding ubiquitin E3 ligase; control of mRNA translation and degradation	[69]
RNAi	*MRE11*	Nuclease; DSB signaling and repair, RF restart	[113]
RNAi	*MUS81*	Nuclease; HR, RF restart, MiDAS	[85,86,97,103,104,106]
RNAi	*PALB2*	HR	[23]
RNAi	*PICH*	DNA translocase; UFB resolution	[108]
RNAi	*PIGN*	Biosynthetic pathway of the glycosylphosphatidylinositol (GPI) anchor	[69]
Inhibitor	*PLK1*	Kinase; cell cycle regulator, control of chromosome segregation	[97]
RNAi	*POLD3*	Replication, BER, MMR, DSBR, NER, MiDAS	[97]
RNAi	*POLH*	TLS, SHM, BER, replication	[87,91]
RNAi	*POLK*	TLS, NER, replication	[92]
RNAi	*RAD18*	TLS	[91]
RNAi	*RAD51*	HR, RF protection	[23]
RNAi	*RAD51C*	RAD51 paralog; HR, RF protection	[117]
RNAi	*RAD52*	ssDNA annealing, backup HR pathway, MiDAS	[100]
RNAi	*RECQ5*	Helicase; HR regulator, RAD51 nucleofilament disruption	[104]
KO	*REV3*	Pol catalytic subunit; TLS, DSBR, FA pathway, SHM	[90]
RNAi	*RIF1*	NHEJ, replication timing	[108]
RNAi	*RPA*	Replication, RS signaling, HR, DNA repair	[23]
Inhibitor	*RRM2*	Ribonucleotide reductase	[116]
RNAi	*SLX1*	Resolvase; HR, RF restart, ICL repair	[103]
RNAi, KO	*SLX4*	Docking platform for nucleases; ICL repair, RF restart, MiDAS	[97,102,103]
RNAi (in G2/M)	*SMC2*	Regulator of chromosome condensation	[97]
KO	*SPARTAN*	Protease; DNA-protein crosslink repair, TLS	[123]
RNAi	*TOP2A*	Topoisomerase; relief of torsional stress, chromatid separation	[23]
RNAi	*TOPBP1*	Initiation of replication, ATR activation, HR regulation	[23,41]
RNAi	*TRIP12*	E3 ubiquitin ligase; proteolysis, regulator of RNF168-mediated chromatin ubiquitylation	[42]
Inhibitor	*UBA1*	E1 ubiquitin ligase; regulator of chromatin ubiquitylation	[111]
RNAi	*UBR5*	E3 ligase; proteolysis, regulator of RNF168-mediated chromatin ubiquitylation	[42]
RNAi	*USP1*	Regulator of TLS, FA pathway, HR	[94,95]
RNAi	*WALP*	Regulator of sister chromatid arm cohesion	[97]
Inhibitor	*WEE1*	Kinase; G2/M checkpoint activation, regulator of replication initiation	[126]
Overexpression	*XPG*	Nuclease; NER	[121]
RNAi	*XRCC2*	RAD51 paralog, HR, RF protection	[117]
RNAi	*XRCC3*	RAD51 paralog, HR, RF protection	[117]
RNAi	*ZNF516*	Transcription activation and repression	[69]

BER: base excision repair, DSBR: double-strand break repair, FA: Fanconi Anemia, ICL: inter-strand crosslink, HR: homologous recombination, KO: knockout, MiDAS: mitotic DNA synthesis, MMR: mismatch repair, NER: nucleotide excision repair, RF: replication fork, RNAi: RNA interference, RS: replicative stress, SHM: somatic hypermutation, SSA: single-strand annealing, TLS: translesion synthesis, UFB: ultra-fine bridge.

**Table 2:** Genes involved in the decrease of 53BP1 NBs formation when misregulated.

Misregulation	Gene	Functions Related to 53BP1 NBs Regulation	References
RNAi	*53BP1*	DSBR mediator	[23]
RNAi; inhibitor	*ATM*	Kinase; DSB signaling	[20,23]
RNAi	*ATR*	Kinase; RS signaling	[41]
RNAi; KO	*ATMIN*	ATM activation	[112]
Overexpression	*CDC6*	Initiation of replication	[38]
KO	*H2AX*	Histone variant; DSB signaling	[20]
RNAi	*NUP153*	Nucleoporin; 53BP1 nuclear import	[111]
Overexpression	*RNASEH1*	R-loop degradation	[45]
RNAi	*SMC2*	Regulator of chromosome condensation	[23]
Overexpression	*WIP1*	PP2C family phosphatase; DDR regulator	[45]

DDR: DNA damage response.

## Abbreviations

53BP1	p53 Binding protein 1
ATM	Ataxia telangiectasia mutated
ATR	Ataxia telangiectasia and Rad3-related
BRCA1	Breast cancer 1
CDC	Cell division cycle
CFS	Common fragile sites
CIN	Chromosomal instability
DDR	DNA damage response
DSB	DNA double-strand break
ERCC1	Excision repair cross-complementation group 1 protein
FA	Fanconi anemia
FANC	Fanconi anemia complementation group
HR	Homologous recombination
MCM	Mini chromosome maintenance
MDC1	Mediator of DNA damage checkpoint protein 1
MiDAS	Mitotic DNA synthesis
MRN	MRE11–RAD50–NBS1 complex
NBs	Nuclear bodies
NHEJ	Non-homologous end-joining
PML	Promyelocytic leukemia
PTF	Proximal sequence element (PSE)-binding transcription factor
RF	Replication fork
RNF	Ring finger protein
RPA	Replication protein A
RS	Replication stress
SPOC1	Survival time associated PHD finger protein in Ovarian Cancer 1
ssDNA	Single-stranded DNA
SUMO	Small ubiquitin-like modifier
TIFs	Telomere dysfunction-induced foci
UFBs	Ultra-fine bridges
XPG	Xeroderma pigmentosum complementation group G protein
XRCC	X-ray repair cross complementing
